# Agrin has a pathological role in the progression of oral cancer

**DOI:** 10.1038/s41416-018-0135-5

**Published:** 2018-06-06

**Authors:** César Rivera, Flávia Silva Zandonadi, Celeste Sánchez-Romero, Ciro Dantas Soares, Daniela Campos Granato, Wilfredo Alejandro González-Arriagada, Adriana Franco Paes Leme

**Affiliations:** 1 Laboratório de Espectrometria de Massas, Laboratório Nacional de Biociências, LNBio, CNPEM, Campinas, Brazil; 2 Departamento de Ciencias Básicas Biomédicas, Facultad de Ciencias de la Salud, Universidad de Talca, Talca, Chile; 3 Faculdade de Odontologia de Piracicaba, Universidade Estadual de Campinas, Piracicaba, Brazil; 4 Facultad de Odontología, Universidad de Valparaíso, Valparaíso, Chile

**Keywords:** Prognostic markers, Predictive markers

## Abstract

**Background:**

The extracellular matrix modulates the hallmarks of cancer. Here we examined the role of agrin—a member of this matrix—in progression of oral squamous cell carcinoma (OSCC).

**Methods:**

We evaluated the immunohistochemical expression of agrin in OSCC and dysplasias. Benign lesions were used as control. In subsequent experiments, we investigated whether the silencing of agrin interferes with tumour expansion both in vitro as well as in vivo. To gain insights into the role of agrin, we identified its protein network (interactome) using mass spectrometry-based proteomics and bioinformatics. Finally, we evaluated the clinical relevance of agrin interactome.

**Results:**

Agrin was elevated in malignant and premalignant lesions. Further, we show that agrin silencing interferes with cancer cell motility, proliferation, invasion, colony and tumour spheroid formation, and it also reduces the phosphorylation of FAK, ERK and cyclin D1 proteins in OSCC cells. In orthotopic model, agrin silencing reduces tumour aggressiveness, like vascular and neural invasion. From a clinical perspective, agrin contextual hubs predict a poor clinical prognosis related with overall survival.

**Conclusions:**

Altogether, our results demonstrate that agrin is a histological marker for the progression of oral cancer and is a strong therapeutic target candidate for both premalignant and OSCC lesions.

## Introduction

Worldwide, head and neck squamous cell carcinoma (HNSCC) affects over 500,000 patients per year.^1^ Oral squamous cell carcinoma (OSCC) represents 95% of all forms of HNSCC.^2^ It is the most common malignancy of the head and neck.^3^ Despite advancements in prevention and multimodality therapies, the prognosis of OSCC patients has remained unfavourable in the past few decades.^4^ A better understanding of cellular and molecular mechanisms that promote the progression of OSCC can help improve our approach to this disease.

The process of cancer progression (i.e. local invasion and metastasis) is characterised by rapid cellular growth accompanied by alterations of the microenvironment of the cancer cells.^5^ OSCC can be presented as a natural history, which originates from nontumourigenic keratinocytes which are chronically exposed to carcinogens, following a hyperplasia, oral epithelial dysplasia (OED; in different degrees) and an invasive carcinoma leading to the generation of metastases.^6^

The extracellular matrix (ECM) modulates the hallmarks of cancer, and changes in its dynamics contribute to tumour progression.^7^ Some components of the ECM, which include heparan sulphate proteoglycans, are frequently overproduced in cancer.^8^ Agrin is one of the main heparan sulphate proteoglycans present in the ECM.

Agrin is a multi-domain protein expressed as either a membrane protein or secreted in the ECM.^9^ Agrin has been shown to act as a sensor in developing oncogenic signals associated with the ECM in hepatic carcinomas.^9^ In the context of OSCC progression, a previous study of our group showed that agrin has high expression in OSCC and has a role on cell migration, adhesion and resistance to chemotherapy,^10^ suggesting that agrin also has an oncogenic role in oral cancer.

Agrin can be proteolytically cleaved which generates bioactive fragments that modulate cellular behaviour.^11^ One of the agrin cleavage products, the C-terminal fragment (hereafter called Ct-agrin), has been shown to be a promising new biomarker for pathological processes, including sarcopenia,^12^ renal dysfunction^13^ and colorectal cancer.^14^ The disintegration of the basement membrane upon local invasion processes can release agrin-processing products, such as Ct-agrin.^14^ Within OSCC are active proteases, such as matrix metalloproteinase (MMP)-3 and neurotrypsin,^15, 16^ which are capable of generating this soluble fragment. Focussing on the dynamics of tumour progression, the Ct-agrin could then help explain the role of agrin in oral cancer.

Despite the findings from the aforementioned studies, the contribution of agrin to cancer progression remains unknown. To better understand the role of this protein in OSCC, we studied the immunohistochemistry (IHC) staining of agrin in benign, premalignant (OEDs) and malignant lesions. In addition, we modulated the expression of agrin in aberrant keratinocytes and evaluated processes and characteristics associated with cancer progression in vitro and in vivo. Considering the potential of Ct-agrin, we identified its binding proteins (interactome) in an OSCC context using mass spectrometry-based proteomics and bioinformatics. Finally, we found that agrin interactome is related with clinical outcomes of head and neck cancers.

## Materials and methods

### General design

In the first experiment, we evaluated agrin immunoexpression on tissue biopsies. The effect of agrin silencing in cancer events was evaluated by in vitro and in vivo (orthotopic model) experiments. We induced the overexpression of secreted agrin fragment and ligands able to bind it were identified by mass spectrometry after protein immunoprecipitation. Once the agrin ligands were identified, we visualise the agrin network and evaluate its potential prognostic value.

### Subjects

#### Patients

We retrospectively collected OSCCs (*n* = 58), OEDs (*n* = 40) and benign lesions (*n* = 35) from two oral pathology services (University of Talca and University of Campinas). The information of patients is provided in Supplementary Table S1.

#### Cells

The following cell lines were used: HMK and HaCaT (nontumourigenic human keratinocytes), SCC-9 and SCC-25 (OSCC), HSC-3 and SCC-9-LN1 (highly invasive and metastatic OSCC cells), and HEK-293 (variable tumourigenic potential). All cells were cultured in recommended media under standard conditions (Supplementary Table S2). HaCaT, SCC9 and HSC-3 were used to establish agrin-silenced cells. The HEK-293 cell line was used to establish cells that overexpress secreted agrin.

#### Mice

Age-matched NOD-SCID male mice (6 weeks old) were obtained under specific pathogen-free conditions (FMUSP, São Paulo, Brazil). Animals were maintained under controlled conditions with freely available food and water, in groups of four mice each.

### Procedures

#### Measuring agrin expression in oral lesions

Immunostaining was performed using the sodium citrate standard protocol for antigen retrieval. Primary antibody used was an agrin antibody (1:300 dilution; #374117, Santa Cruz). Staining was quantified using the IHC profiler plugin in ImageJ.^17^ Additionally, IHC slides were evaluated by two pathologists blinded to clinical data who provided a consensus opinion of staining patterns by light microscopy. According to the consensus, we described intensity (signal strength) and geographical spread. Intensity was classified as 0 = negative, 1 = low, 2 = positive and 3 = high. Geographical spread was classified as 0 = no epithelial staining, 1 = lower third, 2 = two thirds or more and 3 = full thickness. For relative risk (RR) analysis, grades 0–1 were merged into the ‘level 1’ group and grades 2–3 were labelled as ‘level 2’. Briefly, RR corresponded to the proportion of cancer or premalignant lesions in cases with higher expression of agrin divided by the proportion of cancer or premalignant lesions in cases with lower expression.

#### Generation of agrin-silenced cells

We performed agrin (isoform 1, also known as secreted agrin) silencing studies using short hairpin RNA (shRNA)-expressing vectors. We cloned agrin shRNA constructs #TRCN0000056390 and #TRCN0000056391 (RNAi Consortium) into pLKO.1-TRC plasmid (Addgene #10878). We chose the first target for functional experiments (target and template sequences shared a complete alignment). pLKO.1-shGFP was used as control (shControl).^18^ Target sequences are as follows: shAgrin 5′-CCTGCTCTACAACGGGCAGAA-3′ and shControl 5′-CAAGCTGACCCTGAAGTTCAT-3′. Procedures for packaging shRNA-encoding lentivirus were performed at the Viral Vector Laboratory (LNBio-CNPEM, Campinas, Brazil). We generated two cell groups: agrin-silenced cells (shAgrin) and control cells (shControl). Agrin silencing was verified by real-time quantitative PCR (RT-qPCR) and western blot.

#### Generation of C-terminal agrin-overexpressing cells

We simulated a secreted bioactive fragment of agrin using the C-agrin_4,19_-GFP construct^19^ (Ct-agrin; Supplementary Figure 2A). This fragment was kindly gifted by Dr Matthew P. Daniels (NIH, Bethesda, MD, USA). We used as control a FLAG-tagged green fluorescent protein (GFP) (named IP-control) cloned into a pcDNA3 vector (Invitrogen, Thermo Fisher Scientific Inc.). Ct-agrin construction produced cytotoxicity in SCC-9 and HSC-3 cells (Supplementary Figure 2B). This problem was solved by transfecting HEK-293 cells using polyethylenimine (Polysciences Inc.) (Supplementary Figure 2C).

#### Gene expression analysis

RNA isolation and RT-qPCR were performed according to previously published protocol.^20^ Primers used in this study can be found in Supplementary Table S3.

#### Immunoblotting detection

Cell lysate and secretome isolation for western blot analysis were performed as previously described.^20, 21^ Antibodies used are listed in Supplementary Table S4.

### Agrin and cancer-associated events

#### Proliferation

HaCaT, SCC9 and HSC-3 cells either transduced with control shRNA or agrin shRNA (1 × 10^4^ cells/well) were seeded in 96-well plates. Bromodeoxyuridine labelling assay was performed as described.^22^

#### Migration and tumour cell invasion activity

Motility assays were performed as described,^23^ with some modifications. For migration, we used 7.4 × 10^4^ cells/well (HaCaT, SCC-9 and HSC-3) and 24-well chambers with uncoated 8-mm pore polycarbonate membranes. For invasion, we used 8 × 10^4^ cells/well (SCC-9 and HSC-3) and 96-well chambers precoated with Matrigel Basement Membrane Matrix (BD Biosciences).

#### Cancer colony formation

Control or agrin shRNA-tranduced SCC-9 and HSC-3 cells (5 × 10^3^ cells/well) were cultured in 6-well plates. The culture medium was changed every 2 days. After 9 days, cells were stained with 4% formaldehyde/0.005% gentian violet solution. Images were captured with an inverted microscope. We quantified colonies using ImageJ histogram tool (darkest pixels were analysed).

#### Multicellular tumour spheroid formation

To simulate cancer cells in the blood or lymphatic circulation, we performed a three-dimensional tumour sphere culture. Control or agrin shRNA-tranduced SCC9 and HSC-3 cells (6 × 10^5^ cells/dish) were cultured in non-adhesive conditions, as described previously.^24^ The multicellular tumour sphere area was analysed using the ImageJ particle analysis tool.

#### In vivo tumourigenesis and aggressiveness of lesions

We utilised a murine orthotopic model for OSCC. Mice were randomly divided into the following 2 groups (*n* *=* 8 animals in each): HSC-3 shControl and shAgrin. Then 2.5 × 10^5^ cells/tongue in 20 μL of Matrigel were intrabuccally implanted into the right lateral portion of the tongue. Animal health was monitored daily. After 21 days, tumour severity was established by the presence of ulcerations and conventional histopathological examination. We evaluated the growth pattern, keratinisation, cell morphology, angiogenesis and vascular (intravascular tumour thrombus) and neural invasion.

#### Rescue-like experiments

To evaluate whether the specificity of agrin rescues the phenotypes, we exchanged the conditioned media from HSC-3 shControl and shAgrin cells in proliferation, invasion and wound-healing assays. Conditioned media (10 μg) from each group (hereafter called rescue-like medium and shAgrin medium, respectively) was added in serum-free media as described previously.^20^ For wound-healing experiments, 2 × 10^4^ cells/well were used as described previously.^25^

### Determination of agrin-binding partners

#### Immunoprecipitation

We used the secretome extract of Ct-agrin and IP-control cells. Immunoprecipitation was performed at 4 °C with 2.5 μg of GFP antibody (#af4240, R&D Systems) in the presence of 30 µL of protein G-Sepharose 4 Fast Flow (GE Healthcare) for 2 h in a rocker. Sample proteins of 250 μg were added and incubated with the beads overnight at 4 °C. The sepharose-bound proteins were washed with cold TBST. Bound proteins were eluted with 4× Laemmli sample buffer at 95 °C for 10 min and resolved by 10% sodium dodecyl sulphate-polyacrylamide gel electrophoresis (SDS–PAGE) for subsequent western blot analysis.

#### Protein identification

From 3 independent experiments, we excised, reduced, alkylated, trypsin-digested and desalted 60 SDS-PAGE gel bands containing proteins of agrin complexes according to previous protocols^26^ (Supplementary Figure 2D). Tryptic digested peptides were identified in a LTQ Orbitrap Velos mass spectrometer (Thermo Fisher Scientific Inc.) according to previous protocols.^26^ Identification of proteins was performed using the MaxQuant^27^ and Perseus^28^ software, as described previously.^29^ For bioinformatics analysis, we focussed only on proteins identified exclusively in the Ct-agrin group. See Supplementary file for further details and explanation.

### Agrin interactome characterisation

#### Bioinformatics

We used Integrated Pathway Analysis Database for Systematic Enrichment Analysis (IPAD)^30^ to evaluate the inter-association between our identified protein lists and diseases. In IPAD, Fisher Exact test is adopted to measure the gene enrichment in annotation terms and the enrichment between components. IPAD adjust the *P*-value by Benjamini–Hochberg method.^31^ We considered a strong model if oral cancer appeared within the top five ranked epithelial diseases. Agrin-binding partners (Ct-agrin exclusive proteins) were described using FunRich.^32^ Then networks and hubs were visualised using Contextual Hub Analysis Tool (CHAT app) in the Cytoscape software.^33, 34^ To prioritise proteins, we used the cBio cancer genomics portal^35^ and HNSCC-TCGA data set^36^ selecting the genomic profiles by default (mutations, putative copy-number alterations from GISTIC, mRNA Expression *z*-Scores [RNA Seq V2 RSEM] and protein expression *Z*-scores [RPPA]). To explore whether prioritised candidates represent a community, we used the STRING database.^37^ In addition, we evaluated the gene-expression levels of agrin contextual hubs in shControl and shAgrin cells using RT-qPCR. The agrin group also was analysed using SMART^38^ and PAZAR database.^39^

#### Overall survival data

We evaluated the prognostic potential of agrin contextual hubs. Gene expression data in head and neck cancers was obtained from the following publicly available databases: PROGgeneV2^40^ (GSE65858 data set) and SurvExpress^41^ (HNSCC-TCGA provisional data set).

#### Statistics

All independent experiments were performed in triplicate. The results are presented as the mean ± standard deviation (SD). We analysed differences between groups using Chi-Square, Student’s *t*-test, Fisher’s exact test and one-way analysis of variance (ANOVA with post hoc Tukey) tests. In all the procedures, we used a 95% confidence level (*P*-value ≤ 0.05).

## Results

### Agrin is elevated in malignant and premalignant lesions

**Fig. 1:**
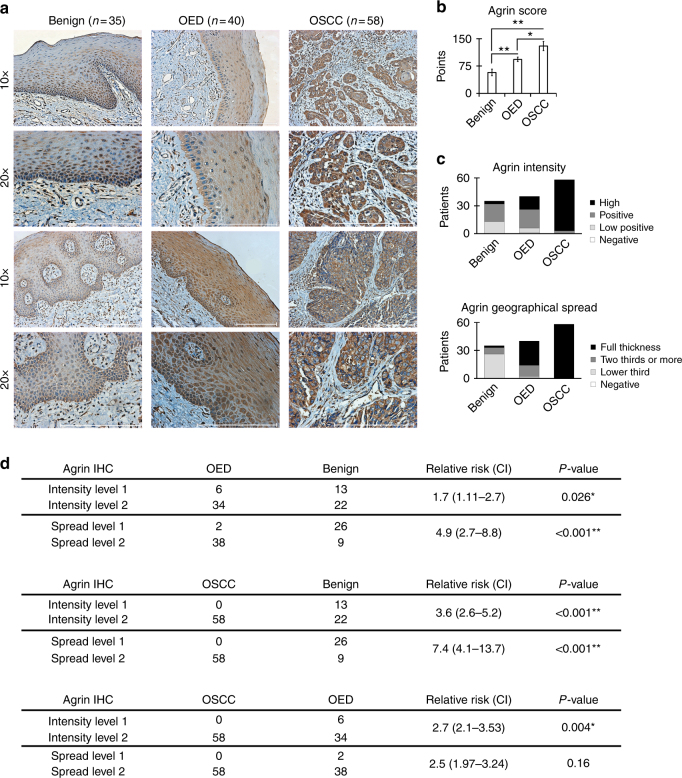
Agrin is overexpressed in oral cancer and epithelial precursor lesions. **a** Representative microphotographs of agrin immunohistochemical staining in oral tissues. Positive staining of agrin is shown in brown. Scale bars, 200 μm. **b** Stronger agrin score staining is observed in oral squamous cell carcinoma (OSCC) and oral epithelial dysplasia (OED) (ANOVA followed by Tukey’s test). Data are present as the mean ± SD (*P-value ≤ 0.05, **≤0.001). **c** Intensity and geographical spread for agrin staining. Premalignant and malignant diagnosis can be correlated with the increase in agrin staining (visualised by black coloured bars). **d** Expression level of agrin was closely associated with patient diagnosis (relative risk; CI confidence interval, Fisher’s exact test). Agrin intensity and geographical spread were dichotomised as levels 1 and 2

Oral cancer is a multi-stage disease.^2^ We believe that its progression can be studied by comparing benign tissues, OEDs and malignant lesions. By IHC, we detected higher expression of agrin in OSCCs (*n* = 58) and OEDs (*n* = 40) than in benign tissues (fibrous hyperplasia and hyperkeratosis, *n* = 35) (Fig. 1a, b). Epithelial staining intensity and reactivity were dichotomised, as shown in Fig. 1c. To further investigate the clinical significance of agrin expression in OSCC progression, we examined the association between agrin staining status and patient diagnosis. The results indicated that high expression of agrin was associated with the presence of OEDs and OSCC (see RRs in Fig. 1d). These results may indicate that, as cells increase agrin expression, the premalignant or malignant changes may become enhanced, shifting the balance from reversible status to tumour progression.

### Agrin silencing suppresses cancer progression events, but conditioned media enriched with Ct-agrin rescue these effects

**Fig. 2:**
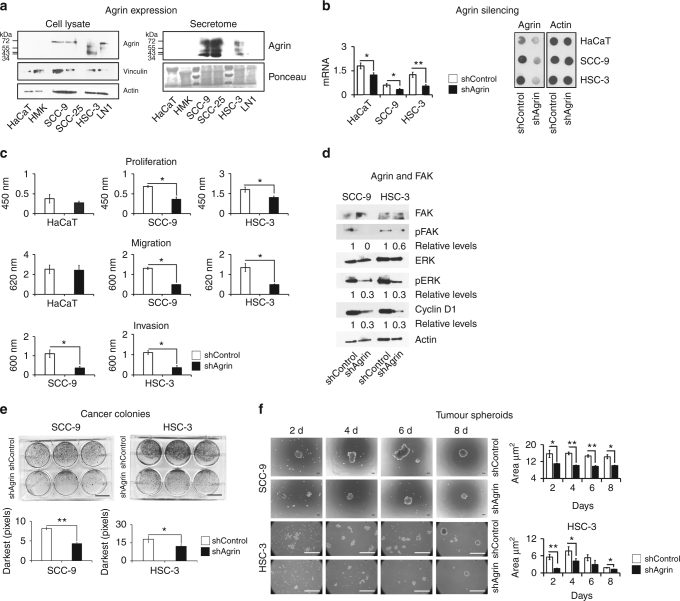
Agrin silencing decreased oral cancer progression in vitro in less (SCC9) and highly (HSC-3) invasive OSCC cells. **a** Western blotting confirmed the presence of agrin in different cell lines both in the cell lysate and secretome. Multiple bands may represent proteolytic cleavage products, besides the alternative splicing variants and posttranslational modifications. Vinculin, actin and ponceau red were used as loading control. **b** Verification of agrin silencing was performed by RT-qPCR and dot blotting. Actin expression was used as an internal control. (**c**) Agrin silenced cells (shAgrin) proliferate, migrate and invade less compared to the control (shControl; absorbance at 450, 600 and 620 nm, respectively). **d** Agrin silencing decreased pFAK, pERK and cyclin D1 protein levels. **e** Focus-formation assay demonstrates less number of colonies in agrin silencing compared to the control. Darkest intensities are represented in millions of pixels. Scale bars, 2 cm. **f** Agrin silencing interferes with tumour sphere formation. Scale bars, 100 µm. Area in µm^2^ (x100,000 to SCC9 and x10,000 to HSC-3). For all RT-qPCR experiments, data were normalised with *GAPDH* gene. Data are represented by the mean ± SD of three independent experiments performed in triplicates (Student’s *t*-test, *P-value ≤ 0.05, **≤0.001)

**Fig. 3:**
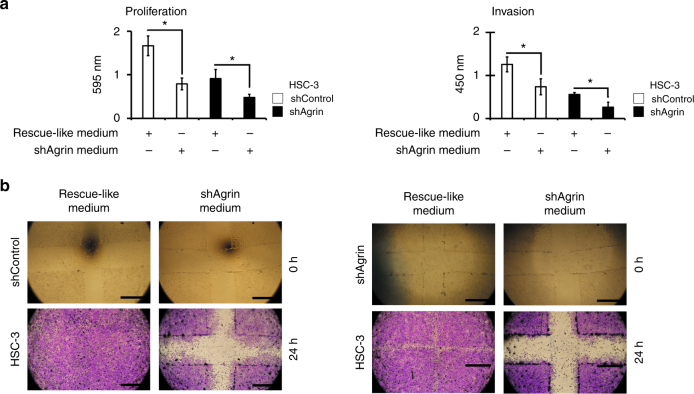
Secretome containing agrin plays a role in cancer progression events. In rescue-like experiments, HSC-3 shControl secretome (rescue-like medium) enhanced proliferation, invasion (**a**) and wound healing (**b**) in shAgrin cells. Data are represented by the mean ± SD of three independent experiments performed in triplicates (*P-value ≤ 0.05 ANOVA followed by Tukey’s test). Scale bars, 0.1 cm

Most oral cancer cell lines secreted high levels of agrin (Fig. 2a, right panel, band having a molecular weight of ~ 72 kDa). We used shRNA technology to knockdown agrin expression in three cell lines. The knockdown efficiency of agrin shRNA was confirmed by RT-qPCR and dot blot (Fig. 2b). Compared to non-target shRNA, treatment with agrin shRNA resulted in a significant decrease in cell proliferation, migration, invasion (Fig. 2c) and *EGFR* mRNA levels (Supplementary Figure 3). Recent studies indicate that agrin provides stimulatory signals to augment focal adhesion kinase (FAK) activity during cancer growth and invasion.^9, 42^ FAK induces cell cycle progression through cyclin D1, involving extracellular-signal-regulated kinase (ERK), among others.^43^ In OSCC cells, treatment with agrin shRNA reduced the levels of FAK and ERK phosphorylated forms, as well as reduced the expression of cyclin D1 (Fig. 2d). In addition, agrin silencing suppresses the colony and tumour spheroid formation (Fig. 2e, f). Taken together, the data suggest that silencing agrin in oral cancer cells results in an impairment of in vitro proliferative and invasive growth programmes. Next, we examined the effect of shControl secretome (conditioned medium) on cell migration, invasion and wound-healing experiments. The results showed that exogenous shControl secretome rescues cell motility in agrin-silenced cells (Fig. 3a, b). Rescue-like medium had a higher capability to stimulate proliferation, invasion and wound healing compared to the secretome originating from shAgrin cells. These results demonstrate that progression of oral cancer may depend, at least in part, on the availability of agrin in the tumour microenvironment.

### Agrin silencing reduces tumour aggressiveness

**Fig. 4:**
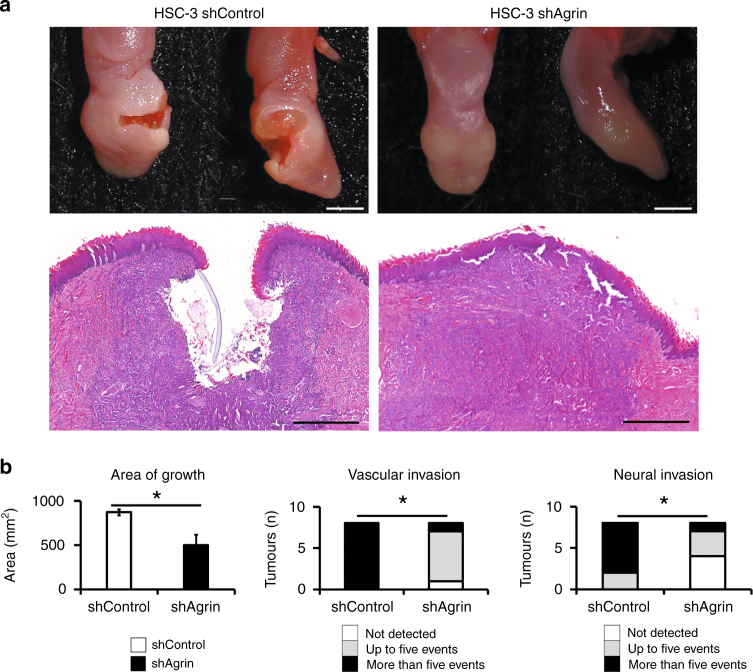
Agrin regulates the aggressiveness of oral cancer. **a** An orthotopic model of OSCC was established, inoculating HSC-3 cells into the lateral border of the tongue of NOD-SCID mice. Animals received control (shControl) or silenced (shAgrin) cells. Representative images are shown (day 21). Scale bars, 0.2 cm and 400 µm (microphotographs). **b** Main histopathological characteristics of oral cancers are demonstrated above (*P-value ≤ 0.05 Student’s *t*-test and Pearson’s Chi-square test)

As shown in Fig. 4a, mice that received agrin-silenced cells (shAgrin) developed less aggressive tumours. These tumours did not have ulcers, instead they showed a well-defined mass tumour formation. According to the histopathological evaluation, shAgrin group produced smaller tumours with few instances of vascular and nervous invasion (Fig. 4b). Tumours generated from control cells show significantly greater aggressiveness in comparison to tumours originating from agrin-silenced cells. An additional panel of histological images can be seen in Supplementary Figure 1.

### Agrin interactome reveals an enrichment in cellular growth process

**Fig. 5:**
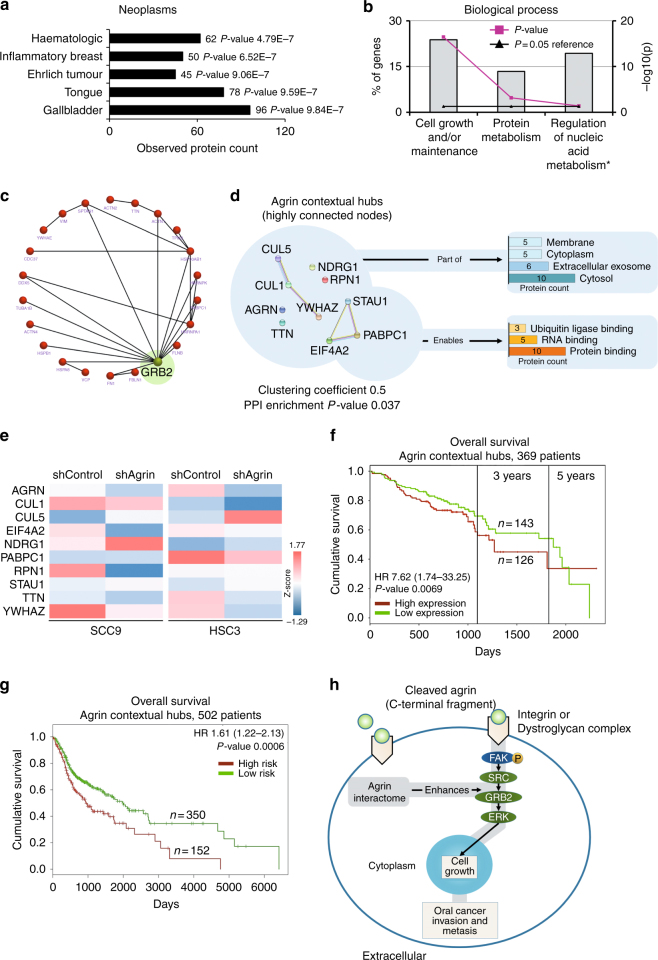
High expression of agrin contextual hubs predict poor prognosis. **a** IPAD predicted diseases with exclusive Ct-agrin ligands as input. Cellular model was strong due to the presence of tongue cancers within the top 5 ranking (top neoplasms from 2,630 diseases). **b** Classification of biological processes of candidate proteins was performed by FunRich. A total of 197 proteins were analysed. Asterisk (*) represents regulation of nucleobase, nucleoside, nucleotide and nucleic acid metabolism. **c** FunRich identifies the interaction between GRB2 (predicted) with many candidates potentially associated with agrin. **d** Prioritised candidates using CHAT app and cBio cancer genomics portal. STRING clustering coefficient and protein–protein interaction (PPI) *P*-value are shown. **e** Agrin silencing in OSCC cells alters gene expression of agrin contextual hubs (data for independent replicates are presented in Supplementary dataset 2). **f**–**g** Analysis of HNSCC patient survival. Tumour samples that exhibit higher expression of agrin interactome show poor patient prognosis. **f**, **g** represent different cohorts. **h** A hypothetical model suggesting the role of agrin in oral cancer: (i) overexpression of secreted or cleaved agrin triggers elevated binding to its receptors promotes FAK activation and (ii) pro-tumourigenic activation of agrin contextual hubs could potentiate this pathway

To better understand how agrin participates in malignant progression, we aimed to identify proteins interacting with Ct-agrin in HEK-293 cells. After the immunoprecipitation experiments, proteins bound to secreted agrin complex were identified by mass spectrometry. 197 proteins were analysed exclusively interacting with agrin complex (Supplementary dataset 1). Since that the interactome of a protein can be highly cell-type dependent, we examined all interacting partners using the IPAD^30^ with focus in malignant diseases. As shown in Fig. 5a, tongue neoplasms are in the top five predicted protein diseases related to agrin partners. We also submitted these candidate proteins to FunRich^32^ tool for functional analysis. We found that cell growth is the major biological category among the agrin-interacting candidates (Fig. 5b). Interestingly, FunRich identifies the interaction between the growth receptor-bound protein 2 factor (GRB2, not identified in our liquid chromatography–tandem mass spectrometric experiments) with many candidates potentially associated with agrin (Fig. 5c). The interaction of GRB2 with FAK and other proteins leads to activation of the Ras and ERK pathways, which induce tumour cell spreading through cytoskeleton rearrangement.^44^

### Prioritisation of agrin-interacting partners reveals the “agrin contextual hubs”

We assigned numeric values to all identified agrin interacting candidates as follows: −3 (Ip-control-exclusive proteins), +3 (Ct-agrin-exclusive proteins), and 1 (common proteins). Networks were visualised using CHAT app. Using a hypergeometric test, CHAT identifies hub nodes that interact with more “contextual” nodes (i.e. Ct-agrin-exclusive proteins) than statistically expected in networks integrated with user-supplied contextual data (e.g. gene-expression data). CHAT term these nodes as contextual hubs. Contextual hubs are considerably more relevant than degree-based hubs to the specific experimental context under investigation.^33^ Neighbour interactors were sourced from four databases (InnateDB-all, Mentha, IntAct and UniProt). *P*-values calculated by CHAT are automatically corrected using the Benjamini–Hochberg procedure. Then we identified the most important centres of activity (the top 20 smallest *P*-values). To prioritise candidates, we used the cBio cancer genomics portal (2016 version).^35^ From that top 20, we chose those proteins that presented any alteration (amplification, deep deletion, mRNA upregulation or downregulation, truncating or missense mutations) in a percentage equal to or higher than 20% of The Cancer Genome Atlas HNSCC sample (TCGA Nature 2015, *n* = 279)^36^ (Supplementary dataset 1). We selected 9 proteins: cullin-1 (CUL1), cullin-5 (CUL5), eukaryotic initiation factor 4A-II (EIF4A2), protein NDRG1 (NDRG1), polyadenylate-binding protein 1 (PABPC1), dolichyl-diphosphooligosaccharide-protein glycosyltransferase subunit 1 (RPN1), double-stranded RNA-binding protein Staufen homologue 1 (STAU1), titin (TTN), and 14-3-3 protein zeta/delta (YWHAZ). Henceforth, we refer agrin and its nine partners as the “agrin contextual hubs” (the function of each member is described in Table 1). Additionally, Supplementary Table S5 shows OncoPrints (distinct genomic alterations, including somatic mutations, copy number alterations and mRNA expression changes) across a subset of OSCC cases.

**Table 1:** Agrin contextual hubs. The function of each member is described below according to literature mining

Protein (gene name)	Changes in mRNA expression^a^	Information
Cullin-1 (CUL1)	(+)	CUL1 and CUL5 provide a scaffold for ubiquitin ligases. They participate in the processes of ubiquitylation and neddylation, which lead to the degradation of tumour-suppressor proteins.^65^ Aberrant expression of CUL1 was found in a number of human cancers that is closely associated with poor patient prognosis^66^
Cullin-5 (CUL5)	(−)	CUL-5 expression is downregulated in breast tumours and its overexpression decreases breast cancer cell growth^67^
Eukaryotic initiation factor 4A-II (EIF4A2)	(+)	Boosts the malignant phenotype in solid tumours^68^
Protein NDRG1 (NDRG1)	(−)	It is associated with a low metastases rate. ^69^ In several cancers, it was suggested to be a tumour-suppressor gene. Decreased expression of NDRG1 is correlated with tumour progression and poor prognosis in patients with oesophageal squamous cell carcinoma.^60^
Polyadenylate-binding protein 1 (PABPC1)	(+)	Can contribute to the aggressiveness of inflammatory breast carcinoma.^70^
Dolichyl-diphosphooligosaccharide-protein glycosyltransferase subunit 1 (RPN1)	(+)	Forms part of the ubiquitin proteasome system. It is a structural component of the proteasome.^71^ RPN1 has a significant association with an aggressive tumour phenotype in breast cancer.^72^
Double-stranded RNA-binding protein Staufen homologue 1 (STAU1)	(−)	Stabilises the mRNA in undifferentiated cells but can mediate its degradation in differentiated cells.^73^ STAU1 overexpression affects mitosis entry and impairs proliferation of transformed cells. Participates in a mechanism of posttranscriptional regulation of gene expression that is linked to cell cycle progression in cancer cells.^74^
Titin (TTN)	(−)	Associated with mesoderm pluripotency in human embryonic stem cells.^75^
14-3-3 protein zeta/delta (YWHAZ)	(−)	Shows high expression in patients with oesophageal cancer, and it is associated with poor clinical prognosis.^76^

The function of each member is described above according to literature mining

^a^Tumourigenic gene expression according to the literature. (+) upregulated/overexpressed, (−) down-regulated/downexpressed

### Agrin contextual hubs can potentially modulate transcription factors

To explore whether prioritised proteins represent a community (group of nodes with common processes, purposes or functions), we used the STRING database.^37^ STRING analysis resulted in a network with a high clustering coefficient (proteins have more interactions among themselves than what would be expected for a random set of proteins of similar size, drawn from the genome) (Fig. 5d). To explore this relationship, we evaluated the gene-expression levels of the agrin contextual hubs in shControl and shAgrin cells. Surprisingly, agrin silencing affected gene expression of different members of the agrin group (Fig. 5e). To explore whether agrin serves as a transcriptional regulator, we used SMART.^38^ According to prediction domain tools, agrin does not contain a DNA-binding domain; however, it does not exclude the possibility that agrin modulates RNA expression, since it can bind to transcription or enhancer factors. Therefore, we have also evaluated whether the transcription factors for the 10 genes overlap, which partially explains the gene-expression regulation that was observed in agrin knockdown condition. Analysing the transcription factors for agrin contextual hubs in the PAZAR database for gene-regulatory information,^39^ we observed that EGR-1 is the transcription factor for nine out of the ten genes in the agrin group (Supplementary dataset 1).

### Agrin contextual hubs represent a community with prognostic potential

Since OSCC is the most common type of malignancy arising from the epithelial cells of the head and neck region,^45^ we evaluated a clinical relevance of agrin contextual hubs in head and neck cancers. We used ProgGene^40^ and SurvExpress^41^ online tools in HNSCC publically available gene-expression databases. High agrin contextual hubs expression was associated with lower overall survival (hazard ratio 7.6, confidence interval 1.7–33.3, *P*-value ≤ 0.05) in a German cohort followed up for >5 years (GSE65858, *n* = 269)^46^ (Fig. 5f). Similarly, in the HNSCC–TCGA provisional (SurvExpress, June 2016) data set (by splitting 502 patients into two maximised risk groups according to their prognostic index), we found that high-risk patients had decreased overall survival (hazard ratio 1.6, confidence interval 1.3–2.7, *P*-value ≤ 0.05) (Fig. 5g). These results suggest that higher expression of agrin contextual hubs is associated with a poor clinical prognosis.

## Discussion

A class of molecules with relevant clinical potential, particularly for HNSCC, is heparan sulphate proteoglycans.^47^ They can be found on the cell surface and soluble in the ECM. Investigation of these molecules as participants in cancer progression is of great importance and reveals complex relationships occurring at the microenvironment, cellular and subcellular levels.^48^ In this study, we show that agrin promotes the progression of oral cancer and that its contextual hubs can predict a poor clinical prognosis.

We found that invasive oral carcinomas and premalignant lesions show a strong expression of agrin compared with benign lesions. We calculated RR to use as strength of association^49^ and biopsies with high agrin staining have 2–7 times the rate of malignant or dysplasia diagnoses compared to samples with low staining. Previous research reported that IHC evaluation of agrin is useful to differentiate benign lesions, dysplasias and hepatocellular carcinomas.^50^ In fact, intriguingly, we observed the presence of cleaved or secreted agrin in oral cancer cell secretomes but not in normal or immortalised cells. In addition, agrin can help distinguish between primary lesions of liver and metastasis with a high sensitivity and specificity.^51^ Oral cancer is a multi-stage disease, generated by sequentially malignant events, from epithelial precursor lesions to invasive carcinoma.^2^ Considering the higher expression of agrin in dysplastic and malignant keratinocytes, and its secretion mainly by cancer cells, it could play a role and/or reflect the process of malignant progression. It was a surprise to identify secreted agrin as a band of ~72 kDa, since the proteolytic results of C-terminal fragment of agrin have molecular weights of ~135, ~ 110, ~90 and ~22 kDa. In a model that evaluated agrin expression in synaptogenesis induced by a traumatic brain injury, the authors found agrin expressed only as species of a molecular weight between 75–55 kDa.^52^ They suggest that proteolysis may not be the major regulator of agrin expression since fragments generated by MMP-3 and neurotrypsin were not observed. Recent evidence shows that agrin is a target of several metalloproteinases, generating protein subfragments that can have diverse regulatory activities.^53^ The presence of ~72 kDa agrin band in OSCC secretome may be due to an intermediate processing of known proteases, a cleavage performed by some other protease, or that some alteration in secreted agrin by aberrant squamous cells causes an atypical processing. The nature of this band should be clarified in future work.

To further explore these findings, here we present evidence demonstrating that agrin silencing interferes with oncogenic cell functions. This is consistent with our previous findings where agrin siRNA knockdown promoted a decrease on OSCC cell migration and adhesion.^10^ Conversely, agrin rescue-like experiments restored proliferative and invasive behaviour in agrin-silenced cells. The secretome is a relevant component for cell–cell communication and the crosstalk between tumour and stroma has a key influence on cancer progression.^20^ Our results suggest that the observed cell phenotype may be due to the presence of agrin in the tumour microenvironment.

Moreover, in the context of liver cancer, agrin promotes proliferation, invasion and oncogenic cellular signalling.^9^ The invasive and proliferative phenotypes constitute fundamental biological activities for the progression of malignant diseases,^54^ and agrin contributes in maintaining these phenotypes. Recent data have shown that FAK pathways are crucial downstream signalling axes of agrin function in liver tumourigenesis.^9^ In our experiments, we found that inhibition of agrin expression decreased pFAK, pERK and cyclin D1 protein levels. This clearly demonstrates the relationship between agrin and cell cycle progression promoted by FAK also in oral cancer cells. Notably, orthotopic tumours produced by agrin-silenced cells exhibited reduced aggressiveness, showing less vascular and neural invasion, which are associated with a better clinical prognosis.^3, 55^

We used an immunoprecipitation-based proteomic approach to identify partners of secreted agrin. We discovered 197 proteins potentially interacting with agrin complex. These proteins are mainly related with cell growth, RNA and ubiquitin ligase-binding processes. A close examination using FunRich tool indicated GRB2 (a member of FAK signalling pathway) as an interactor with many proteins potentially presented in agrin complex. It is evident that, in OSCC cells, agrin can be a secreted or cleaved molecule, and as a soluble fragment, agrin can bind to integrins or dystroglycan complex and then could activate FAK,^56^ which induces cell growth, invasion and metastasis through GRB2, SRC, ERK and cyclin D, among others.^43^ Our contextual analysis of potential agrin-binding partners, immunopurified from the extracellular space, identified highly connected nodes located in the intracellular compartment. Within these nodes, we prioritised nine hubs to construct the agrin contextual hubs: CUL1, CUL5, EIF4A2, NDRG1, PABPC1, RPN1, STAU1, TTN, and YWHAZ. These proteins may interact directly or indirectly with secreted agrin. Agrin contextual hubs could interfere and enhance the FAK signalling pathway (a proposed model is presented in Fig. 5h). For example, CUL1 is necessary for the expression of SRC family kinases and FAK.^57^ Conversely, a low expression of CUL5^58^ and NDRG1^59^ allows SRC and FAK activation.

According to our bioinformatics analysis, agrin contextual hubs represent a biologically connected community. This was confirmed by changes in the expression profile of network members when agrin was silenced. For example, an increased expression of NDRG1 was observed in shAgrin cells compared to control cells. Interestingly, NDRG1 lower expression has been previously correlated with tumour progression and poor prognosis in patients with solid tumours.^60^ Then we further investigated how agrin expression affected gene expression of its contextual hubs. Surprisely, we observed using PAZAR database analysis that EGR-1 is a common transcription factor among all the members of the agrin group. In addition to influencing agrin expression, EGR-1 is involved, either directly or indirectly, in the process of agrin cleavage.^61^ Agrin is known to activate FAK and it is a critical regulator of YAP/TAZ function.^62^ A recent work indicates that YAP/TAZ are major oncogenes associated with OSCC.^63^ Since EGR-1 is a factor that also regulates the transcription of YAP and TAZ (according to PAZAR database), it may be interesting to explore whether EGR-1 silencing affects tumour progression or if agrin depletion affects YAP/TAZ activity in oral cancer cells. In this way, further investigations are needed to elucidate the underlying mechanisms of agrin contextual hubs.

In this research, the histopathological data cumulatively support the pathophysiological role of agrin in oral cancer progression. On the clinical perspective, we demonstrated that patients with HNSCC who show a high gene expression of agrin contextual hubs have a lower survival rate. Since agrin is a gateway for a set of proteins with clinical relevance, it is plausible to think that agrin could be a potential therapeutic alternative for future research. In conclusion, our results underscore agrin expression as a novel marker for malignant and premalignant oral lesions and indicates agrin contextual hubs as a prognostic signature for head and neck cancers.

## Electronic supplementary material

Supplementary file

Supplementary dataset 1

Supplementary dataset 2
