# Supplementary material for: Agrin has a pathological role in the progression of oral cancer
Source: Br J Cancer. 2018 Jun 6;118(12):1628–38. doi: 10.1038/s41416-018-0135-5 (PMC6008410; doi:10.1038/s41416-018-0135-5)
Supplement: Supplementary file 1 — Supplementary file [file 41416_2018_135_MOESM1_ESM.docx]

**SUPPLEMENTARY INFORMATION**

**AGRIN HAS A PATHOLOGICAL ROLE IN THE PROGRESSION OF ORAL CANCER**

Rivera, C; Zandonadi, FS; Sánchez-Romero, C; Dantas, C; Granato, DC; González-Arriagada, WA; Paes Leme, AF.

^*^Correspondence**:** Adriana Franco Paes Leme, email: adriana.paesleme@lnbio.cnpem.br, Brazilian Biosciences National Laboratory, LNBio, CNPEM, 13083-970 Campinas, Brazil

**SUPPLEMENTARY METHODS**

**Mass spectrometry and data analysis.** Tryptic-digested peptides were dried in a speed-vac instrument. The samples were analysed on a LTQ Orbitrap Velos mass spectrometer (Thermo Fisher Scientific Inc., Waltham, MA*,*USA) connected to nanoflow liquid chromatography (LC-MS/MS) by an EASY-nLC system (Proxeon Biosystem, Thermo Fisher Scientific Inc.) through a Proxeon nanoelectrospray ion source as described previously. The raw files were processed using the MaxQuant and the MS/MS spectra were searched using the Andromeda search engine against the Uniprot Human Protein Database (UniProt release 2016_03, March 17th, 2016) with a tolerance of 20 ppm for precursor ions and 1 Da for fragment ions. A maximum of 2 trypsin missed cleavage was as set parameter for protein identification. Acetylation and oxidation were set as variable modifications, and carbamidomethylation was set as a fixed modification. Protein intensity values were normalized using the label-free quantification (LFQ) algorithm available through the MaxQuant program. It was used a 2 min window for matching between runs and maximum 1% peptide and 1% protein false discovery rate. Statistical analysis of the data was performed using Perseus version1.5 software. Protein identification datasets were pre-processed for the exclusion of contaminant entries, reverse sequences identification, and only identified by site entries. LFQ intensity values were log2 transformed. The minimum valid values filter was set to 2 in at least one group. Three independent experiments were group in Ct-agrin and IP-control and a paired t-test was applied for testing of differences in protein intensities between these groups.

**SUPPLEMENTARY FIGURES**

**
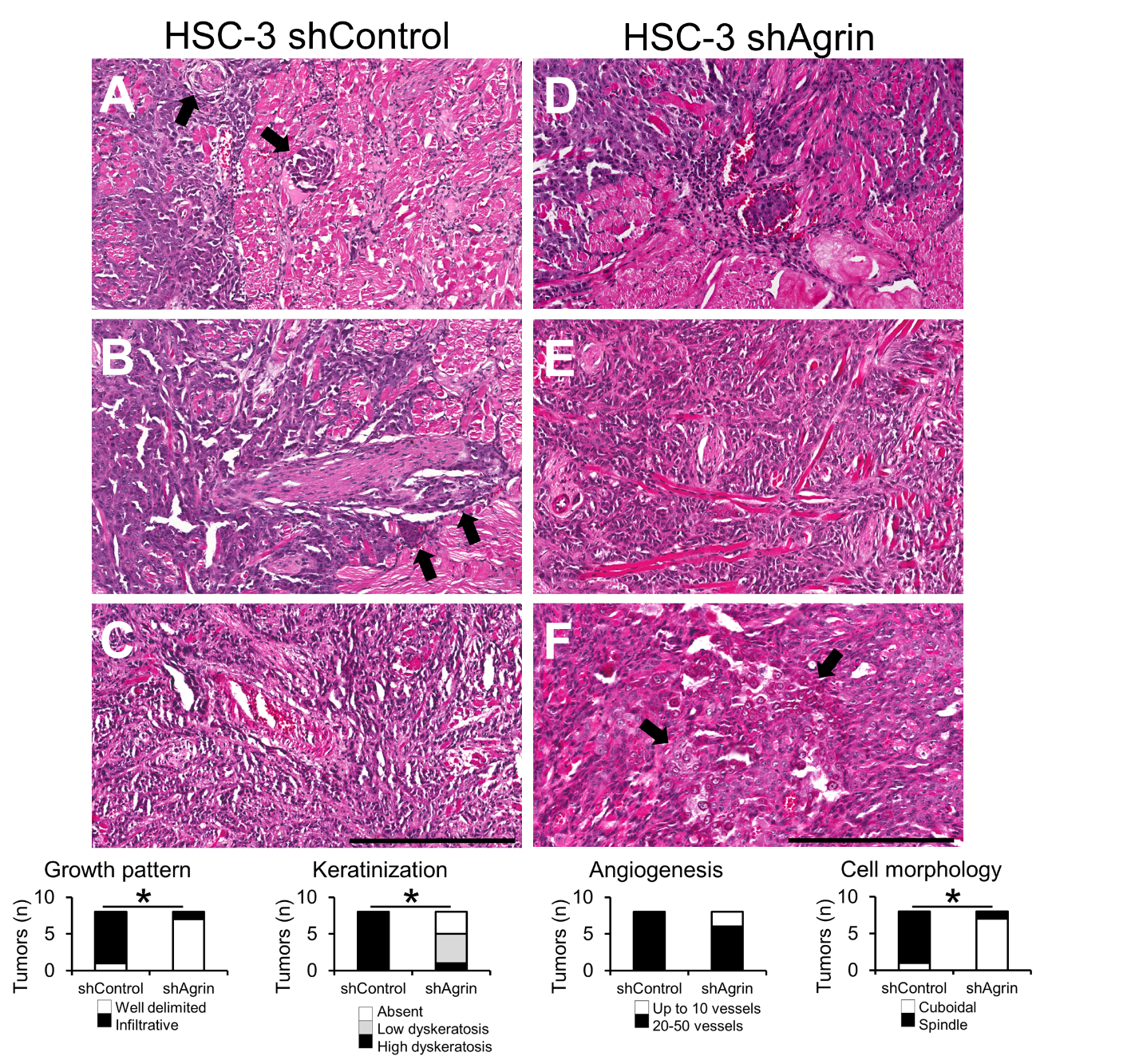
**

**Supplementary Figure 1. Histological features of tongue tumors.**  An orthotopic model of OSCC was established by inoculating HSC-3 cells into the lateral border of the tongue of NOD-SCID mice. (**A-C**) Tumors of HSC-3 shControl group showed an infiltrative growth pattern with discrete/absent inflammatory infiltrate. (**A**) Neural and (**B**) vascular invasion are common findings (arrows). (**C**) Cells demonstrated a spindle cell morphology. (**D-F**) HSC-3 shAgrin tumors showed well delimited growth, with muscular infiltration with moderate/intense inflammatory infiltrate. (**D**) Neural and (**E**) vascular invasion are uncommon findings. (**F**) Neoplastic cells demonstrated a cuboidal morphology and focal keratinization (arrows). Scale bars, 200 µm. Lower panel shows the comparison between both groups (**P*-value ≤ 0.05 Pearson's Chi-square test).


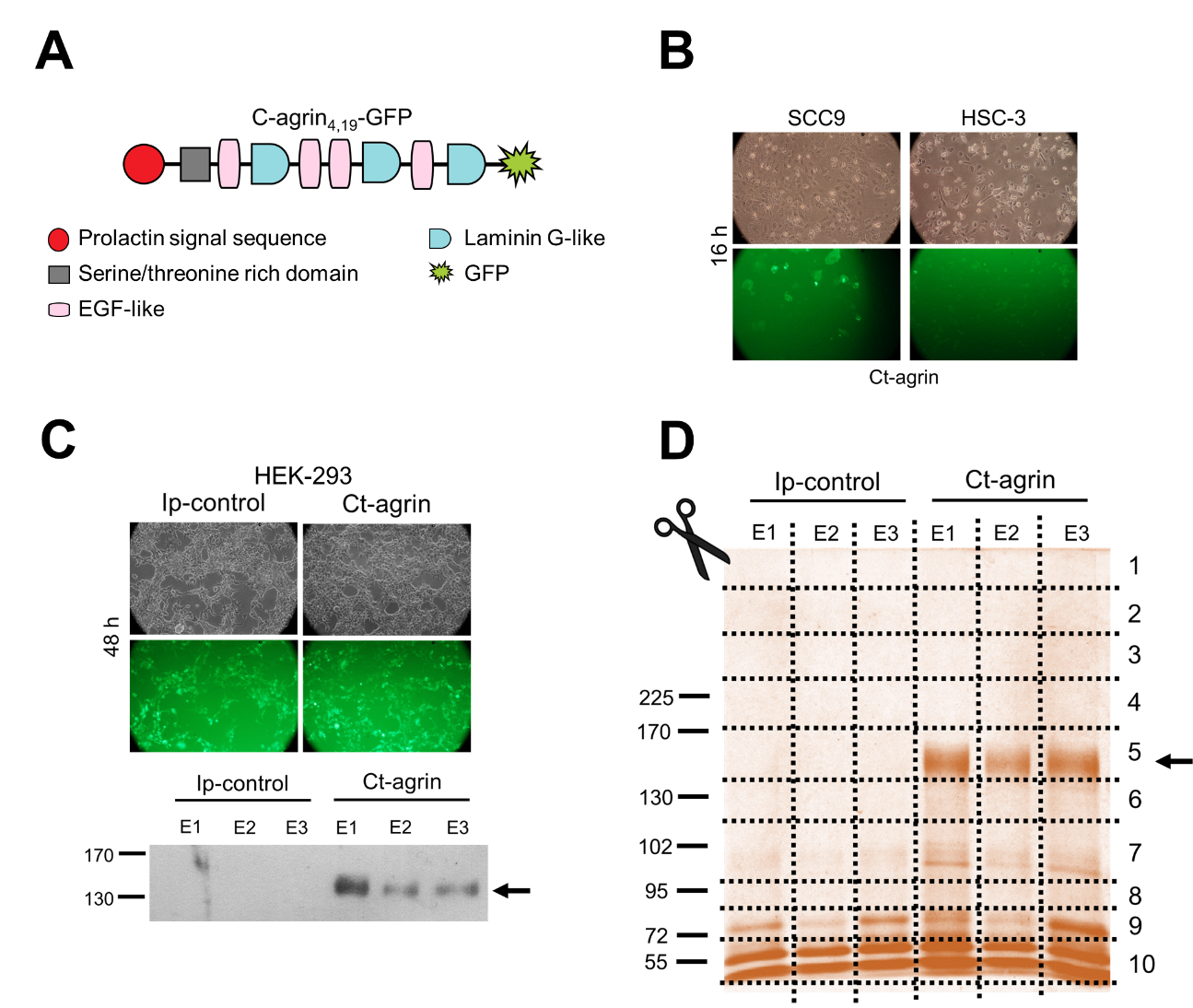


**Supplementary Figure 2. Identification of C-terminal agrin partners.** (**A**) C-terminal agrin-GFP construction was used as Ct-agrin in transfection experiments. (**B**) Non-viral vector transfection induced cell cytotoxicity in OSCC cells. (**C**) Overexpression of secreted protein (arrow) fragment in HEK-293 cells can be visualized by western blot. E1-E3 refer to 3 independent anti-GFP IP experiments that were done to identify Ct-agrin ligands (**D**) Ligands able to bind to Ct-Agrin (arrow) were silver stained in a 10% SDS-PAGE gel and the corresponding lanes were excised and analyzed by mass spectrometry. MaxQuant and Perseus were used for data analysis.


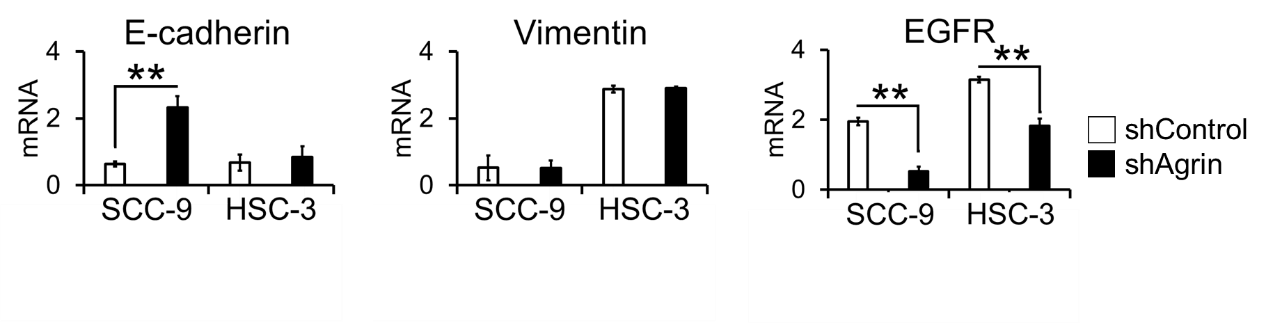


**Supplementary Figure 3.** Some expression factors involved in epithelial-mesenchymal transition are regulated by agrin expression.

**SUPPLEMENTARY TABLES**

**Supplementary Dataset 1 and 2.** Provided as XLSX files.

| **Dataset 1** |  |
| --- | --- |
| **Sheets** | **Title** |
| 1 | Ct-agrin identified proteins |
| 2 | IPAD validation |
| 3 | CHATapp and Cbio portal |
| 4 | PAZAR database |

| **Dataset 2** |  |
| --- | --- |
| **Sheets** | **Title** |
| 1 | Figure qRT-PCR |
| 2 | Z-score |
| 3 | Means |

**Supplementary Table S1.** Distribution of clinicopathological features in the study population.

| **Categories** | **Subcategories** | **Female** | **Male** | **Missing (gender)** | **Total** |
| --- | --- | --- | --- | --- | --- |
| Diagnosis (n=133) | Hyperkeratosis | 3 | 1 | 0 | 4 |
|  | Fibrous Hyperplasia | 18 | 11 | 2 | 31 |
|  | Dysplasia | 28 | 18 | 2 | 48 |
|  | OSCC | 22 | 34 | 2 | 58 |
|  | *Total* | 63 | 64 | 6 | 133 |
| TNM* (n=56) | T1 | 5 | 4 | - | 9 |
|  | T2 | 6 | 11 | - | 17 |
|  | T3 | 4 | 9 | - | 13 |
|  | T4 | 7 | 10 | - | 17 |
|  | *Total* | 22 | 34 | - | 56 |
|  | N0 | 13 | 21 | - | 34 |
|  | N1 | 3 | 6 | - | 9 |
|  | N2 | 5 | 5 | - | 10 |
|  | N3 | 1 | 2 | - | 3 |
|  | *Total* | 22 | 34 | - | 56 |
| Stage (n=56) | I/II | 6 | 11 | - | 17 |
|  | II/IV | 16 | 23 | - | 39 |
|  | *Total* | 22 | 34 | - | 56 |
| WHO differentiation degree  (n=56) | Well | 9 | 15 | - | 24 |
|  | Moderately | 10 | 14 | - | 24 |
|  | Poorly | 3 | 5 | - | 8 |
|  | *Total* | 22 | 34 | - | 56 |
| Pattern of tumor invasion  (n=56) | Cohesive | 9 | 18 | - | 27 |
|  | Infiltrative | 13 | 16 | - | 29 |
|  | *Total* | 22 | 34 | - | 56 |
| Survival status (n=56) | Alive | 9 | 19 | - | 28 |
|  | Deceased | 13 | 15 | - | 28 |
|  | Total | 22 | 34 | - | 56 |

*All patients were M0.

**Supplementary Table S2.** Cell lines used in this study.

| **Cell line** | **Information** |
| --- | --- |
| HMK. Normal, non-tumorigenic. J Oral Pathol Med. 2016;45(9):704-11.  Kindly donated by Dr. Tuula Salo, University of Helsinki, Helsinki. | Human oral mucosal keratinocytes obtained from surgical gingival biopsy and spontaneously immortalized. ***Culture conditions*.** Keratinocyte serum-free medium (Gibco, Paisley, UK) supplemented with antibiotics, 0,005µg/ml recombinant EGF, 0,5mg/ml bovine pituitary extract and 100µM of CaCl_2_. ***Used as*.** Control group (RT-qPCR and WB initial experiments). ***Generated subgroups*.** None. |
| HaCaT. Normal, non-tumorigenic. J Invest Dermatol. 1997;108(1):78-82. | Non-tumorigenic keratinocytes originated from adult human skin. ***Culture conditions*.** DMEM (High Glucose, Sodium Pyruvate, L-glutamine and Phenol Red; Gibco) containing 10% FBS and antibiotics. ***Used as*.** Control group (RT-qPCR, WB, transduction, and functional experiments). ***Generated subgroups*.** HaCaT shControl and shAgrin groups. |
| SCC-9. Oral squamous cell carcinoma (OSCC). Cancer Res. 1981;41(5):1657-63.  American Type Culture Collection, Manassas, VA, USA (ATCC). | Originated from human squamous cell carcinoma from the tongue. ***Culture conditions*.** DMEM/Ham's F12 medium (Cultilab, Campinas, SP, Brazil), supplemented with 10% fetal bovine serum (FBS), antibiotics and 0.4 μg/mL hydrocortisone. ***Used as*.** OSCC group (RT-qPCR, WB, agrin-silencing and functional experiments). ***Generated subgroups*.** SCC-9 shControl and shAgrin groups. |
| SCC-25. OSCC. Cancer Res. 1981;41(5):1657-63.  (ATCC). | Originated from human squamous carcinoma from the tongue. ***Culture conditions*.** DMEM/Ham's F12 with 10% FBS, antibiotics and hydrocortisone. ***Used as*.** OSCC group (RT-qPCR and WB initial experiments). ***Generated subgroups*.** None. |
| HSC-3. Metastatic OSCC. J Oral Maxillofac Surg. 2007;65(9):1725-33.  Japan Health Sciences Foundation, Tokyo, Japan. | Human oral squamous cell carcinoma cell with high metastatic potential, from tongue. ***Culture conditions*.** DMEM/Ham's F12 with 10% FBS, antibiotics and hydrocortisone. ***Used as*.** Metastatic OSCC group (RT-qPCR, WB, agrin-silencing and functional experiments). ***Generated subgroups*.** HSC-3 shControl and shAgrin groups. |
| SCC9-LN1. Metastatic OSCC. Mol Cancer Ther 13:585–595.  Kindly donated by Dr. Edgard Graner, UNICAMP Brazil. | The cell line was originated from SCC-9 cells isolated from mice lymph nodes (LN-1). ***Culture conditions*.** DMEM/Ham's F12 with 10% FBS, antibiotics and hydrocortisone. ***Used as*.** Metastatic OSCC group (RT-qPCR and WB initial experiments). ***Generated subgroups*.** None. |
| HEK293. Unstable karyotype, variable tumorigenic potential. Gene. 2015;569(2):182-90.  (ATCC). | Human embryonic kidney cells. After 65 passages it can form tumors in mice. ***Culture conditions*.** DMEM with 10% FBS and antibiotics. ***Used as*.** Experimental group (Generation of agrin-overexpressing cells and immunoprecipitation experiments). ***Generated subgroups*.** HEK-293 IP-control and C-terminal agrin groups. |

*All cells were maintained at 37°C in a 5% CO_2_ atmosphere. RT-qPCR, real-time quantitative PCR; WB, western blot; sh, short hairpin RNA; IP, immunoprecipitation.

**Supplementary Table S3.** Expression primers used in this research.

| **Name** | **Gene** | **Forward** | **Reverse** |
| --- | --- | --- | --- |
| Glyceraldehydes-3-phospate dehydrogenase | *GADPH* | 5′-GAAGGTGAAGGTCGGAGTCAAC-3′ | 5′-CAGAGTTAAAAGCAGCCCTGGT-3′ |
| Agrin | *AGRN* | 5′-TTGTCGAGTACCTCAACGCT-3′ | 5′-CAGGCTCAGTTCAAAGTGGT-3′ |
| E-cadherin | *CDH1* | 5′-ACAGCCCCGCCTTATGATT-3′ | 5′-TCGGAACCGCTTCCTTCA-3′ |
| Vimentin | *VIM* | 5′-GGCTCGTCACCTTCGTGAAT-3′ | 5′-TCAATGTCAAGGGCCATCTTAA-3′ |
| Cullin-1 | *CUL1* | 5′-CGCTGGCTTTGTGGCTGCTC-3′ | 5′-TGTGGCGGCTGGCGTAGAA-3′ |
| Cullin-5 | *CUL5* | 5′-GAGTGGCTAAGAGAAGTTGGTATG-3′ | 5′-TCTTCTCTCATCCTTTCTGTAGTG-3′ |
| Dolichyl-diphosphooligosaccharide--protein glycosyltransferase subunit 1 | *RPN1* | 5′-CACCCTCAACAGTGGCAAGAAG-3′ | 5′-TGCATTTCGCTCACTCTGTCG-3′ |
| Double-stranded RNA-binding protein Staufen homolog 1 | *STAU1* | 5′-TTTGTGACCAAGGTTTCGGTTGGG-3′ | 5′-TGGGCTTGTCTGTGGCTTGACTAT-3′ |
| Eukaryotic initiation factor 4A-II | *EIF4A2* | 5′-TTTTCGGATCATGTCTGG-3′ | 5′-CAACTGTTGCAGGATGGA-3′ |
| Polyadenylate-binding protein 1 | *PABPC1* | 5′-AGCAAATGTTGGGTGAACGG-3′ | 5′-ACCGGTGGCACTGTTAACTG-3′ |
| Protein NDRG1 | *NDRG1* | 5′-GTGGTTGGGGACAGCTCGC-3′ | 5′-CAGCAGCACCCGAGTTGGGG-3′ |
| Titin | *TTN* | 5′-TCTATGATCGTTTTTGTGATACACGAA-3′ | 5′-GAGCAAAGTGTAACGGCCAACA-3′ |
| 14-3-3 protein zeta/delta | *YWHAZ* | 5′-ATGTACTTGGAAAAAGGCCG-3′ | 5′-CCCTGCTCTTGAGGAGCTTA-3′ |

**Supplementary Table S4.** List of antibodies used in this study.

| **Antigen** | **Source** | **Dilution** | **Use*** |
| --- | --- | --- | --- |
| Agrin | #sc-374117, Santa Cruz Biotechnology | 1:300 | IHC |
| Agrin | #sc-374117, Santa Cruz Biotechnology | 1:500 | WB |
| Actin | #ab3280, Abcam | 1:2,000 | WB |
| Vinculin | #ab18058, Abcam | 1:1,000 | WB |
| Green fluorescent protein (GFP) | #af4240, R&D Systems | 1:10,000 | WB |
| FAK | #sc-558, Santa Cruz Biotechnology | 1:1,000 | WB |
| Phospho-FAK | #44624-G, ThermoFisher Scientific | 1:1,000 | WB |
| ERK | #sc-153, Santa Cruz Biotechnology | 1:1,000 | WB |
| Phospho-ERK | #sc-7383, Santa Cruz Biotechnology | 1:1,000 | WB |
| Cyclin D1 | #sc-374117, Santa Cruz Biotechnology | 1:500 | WB |
| Green fluorescent protein (GFP) | #af4240, R&D Systems Inc | 2.5 μg | IP |

*Recommended secondary antibodies were used. IHC, immunohistochemistry; WB, western blot; IP, immunoprecipitation.

**Supplementary Table S5.** Agrin contextual hubs in OSCC samples* (TCGA provisional dataset, n=320, February 2018).

| **TCGA information** | **Gene name** | | | | | | | | | |
| --- | --- | --- | --- | --- | --- | --- | --- | --- | --- | --- |
|  | **AGRN** | **CUL1** | **CUL5** | **EIF4A2** | **NDRG1** | **PABPC1** | **RPN1** | **STAU1** | **TTN** | **YWHAZ** |
| **Alterations (n=320)** |  |  |  |  |  |  |  |  |  |  |
| Without alterations | 311 (97.2) | 249 (77.8) | 271 (84.7) | 238 (74.4) | 225 (70.3) | 239 (74.7) | 258 (80.6) | 258 (80.6) | 189 (59.1) | 200 (62.5) |
| With alterations | 9 (2.8) | 71 (22.2) | 49 (15.3) | 82 (25.6) | 95 (29.7) | 81 (25.3) | 62 (19.4) | 62 (19.4) | 131 (40.9) | 120 (37.5) |
| **Genetic profile (n=274)** |  |  |  |  |  |  |  |  |  |  |
| **CNA** |  |  |  |  |  |  |  |  |  |  |
| Amplification | 3 (33.3) |  | 1 (2) | 44 (53.7) | 29 (30.5) | 22 (27.2) | 9 (14.5) |  | 10 (7.6) | 22 (18.3) |
| Deep deletion | 2 (22.2) | 4 (5.6) | 4 (8.2) |  |  |  |  | 1 (1.6) |  |  |
| **Mutation** |  |  |  |  |  |  |  |  |  |  |
| Missense mutation | 3 (33.3) | 3 (4.2) | 2 (4.1) | 1 (1.2) | 1 (1.1) |  | 2 (3.2) | 2 (3.2) | 98 (74.8) | 1 (0.8) |
| Truncating mutation | 1 (11.1) | 2 (2.8) | 1 (2) |  |  |  |  |  | 17 (13) |  |
| Inframe mutation |  |  |  |  |  |  |  |  | 1 (0.8) |  |
| **mRNA** |  |  |  |  |  |  |  |  |  |  |
| Downregulation |  | 37 (52.1) | 26 (53.1) |  |  |  |  | 9 (14.5) |  |  |
| Upregulation |  | 25 (35.2) | 15 (30.6) | 37 (45.1) | 65 (68.4) | 59 (72.8) | 51 (82.3) | 50 (80.6) | 5 (3.8) | 97 (80.8) |
| **Survival (n=274)** |  |  |  |  |  |  |  |  |  |  |
| Living | 5 (55.6) | 33 (46.5) | 27 (55.1) | 36 (43.9) | 45 (47.4) | 41 (50.6) | 34 (54.8) | 33 (53.2) | 59 (45) | 69 (57.5) |
| Deceased | 4 (44.4) | 38 (53.5) | 22 (44.9) | 46 (56.1) | 50 (52.6) | 40 (49.4) | 28 (45.2) | 29 (46.8) | 72 (55) | 51 (42.5) |
| Survival (months) | 18.7±15.2 | 33.4±21.7 | 25±17.6 | 26.6±22.8 | 30±25.5 | 32.5±27.3 | 30.2±19.1 | 32.4±21.6 | 30.7±22.1 | 35.4±29.2 |
| Disease free | 3 (42.9) | 32 (72.7) | 23 (60.5) | 26 (51) | 37 (52.9) | 33 (54.1) | 23 (54.8) | 26 (59.1) | 47 (54) | 55 (61.1) |
| Recurred/progressed | 4 (57.1) | 12 (27.3) | 15 (39.5) | 25 (49) | 33 (47.1) | 28 (45.9) | 19 (45.2) | 18 (40.9) | 40 (46) | 35 (38.9) |
| Disease free (months) | 16.3±16.3 | 33.3±21.8 | 21.8±18 | 24.7±23.6 | 27.5±26.8 | 29.4±25.5 | 27.2±20.1 | 30.4±21.9 | 27.5±22.9 | 31.9±28.1 |

Since 2017, cbioportal (<http://www.cbioportal.org/>) allows export cases according to specific anatomical sites (Oncoprint > add clinical tracks). *OSCC includes: Alveolar ridge, buccal mucosa, floor of mouth, hard palate, lip, oral cavity and oral tongue. Parentheses indicate percentages within each gene. From 320 OSCC patients, 274 presented alterations. It is not possible perform a Cox regression, because exported variables are qualitative.
